# Editorial: Nuclear Genome Stability: DNA Replication, Telomere Maintenance, and DNA Repair

**DOI:** 10.3389/fcell.2022.875749

**Published:** 2022-03-10

**Authors:** Marcelo S. da Silva, Richard McCulloch, Maria Isabel N. Cano

**Affiliations:** ^1^ DNA Replication and Repair Laboratory (DRRL), Department of Chemical and Biological Sciences, Biosciences Institute, São Paulo State University (UNESP), Botucatu, Brazil; ^2^ The Wellcome Centre for Integrative Parasitology, Institute of Infection, Immunity and Inflammation, University of Glasgow, Glasgow, United Kingdom; ^3^ Telomeres Laboratory, Department of Chemical and Biological Sciences, Biosciences Institute, São Paulo State University (UNESP), Botucatu, Brazil

**Keywords:** DNA replication, DNA damage response, DNA repair, genome stability, telomere regulation, noncoding RNAs, R-loops

## DNA Replication, Replication Stress, and Genome Instability

DNA replication is an essential and tightly regulated process that follows several ordered steps occurring at the S phase of the cell cycle. Replication is triggered after replication origins are licensed at the G1 cell cycle phase. Once origins are activated, specific DNA helicases open the double-stranded DNA promoting the formation of bidirectional replication forks, allowing replication initiation per se. During semi-conservative replication, DNA polymerases copy the parental strands using RNA as a primer. DNA synthesis is continued by the addition of dNTPs to the 3′ end of the growing strands, ensuring the reliable replication of both DNA strands. However, tight DNA-protein complexes can slow down replication, inducing fork pausing/stalling, which is an active process involving the recognition of a protein barrier by the approaching replisome (via the Fork Pausing/Protection Complex, FPC). This evolutionarily conserved protein complex avoids fork collapse, promoting genome integrity. Shyian and Shore compiled current knowledge about DNA replication pausing in eukaryotes and reminded that fork pausing could also be accidental, and it can also be programmed for various purposes. The authors reviewed the growing number of approaches used to study DNA replication pausing *in vivo* and *in vitro* and new factors involved in modulating fork pausing in different systems. They emphasized the role of Topoisomerase I and II, which slow down replication forks at protein barriers either by direct inhibition of CMG helicase or indirectly by preventing the build-up of barrier-disrupting DNA topology. Therefore, they proposed barrier models where replisome recognizes either non-specific barriers, such as supercoiled DNA, or specific barriers formed by the interaction between the proteins in the barrier and replisome. They also commented on how barriers prevent replication-transcription collisions avoiding double-strand breaks (DSBs) and consequent genome instability. Moreover, they highlighted the absence of enough knowledge about fork pausing in humans and conservation.

Hamadeh and Lansdorp reviewed the role of RECQL5 in resolving intermediate DNA repair structures resulting from the collision between replication and transcription. DNA replication and transcription are challenging for genome integrity since these important cellular events use DNA as substrate. RECQL5 belongs to a class of helicases encoded by five different genes (RECQL1, BLM, WRN, RECQL4, and RECQL5) unique to mammals. There is only one homolog, RecQ, in single-celled eukaryotes and bacteria. Among these helicases, only BLM, WRN, and RECQL4 were associated with rare genetic disorders and predisposition to cancer. However, similar to other RecQ deficiencies, RECQL5 in mice was associated with cancer development and in human cells, to chromosome instability, elevated sister chromatid exchange, and DSBs. But a vast number of investigations show that the ability of RECQL5 to resolve intermediate DNA repair structures is probably associated with its unique C-terminal domain that consists of multiple protein-protein interaction motifs. For example, RECQL5 can associate with RAD51 filaments in different cell scenarios. It can also interact with PCNA, RNA polymerase II, and other proteins involved in DNA replication, repair, and transcription, arguing in favor of RECQL5 being an important regulator of genome integrity.

Nguyen et al. discussed the role of OB-fold proteins (replication protein A–RPA; breast cancer susceptibility protein 2—BRCA2, and the components of CST complex–CTC1, STN1, and TEN1) in replication stress. Proteins containing OB-fold (oligonucleotide/oligosaccharide) binding domains, show high affinity for single-stranded DNA (ssDNA). At the replication fork, they protect ssDNA from nuclease attack and reannealing. Among these proteins, RPA is one of the best-studied. It protects ssDNA from nucleolytic degradation, forming a platform that helps recruit different binding partners. During replication stress, the binding of RPA to ssDNA at a stalled fork or resected DSB can 1) recruit the ATR-ATRIP (ATR-interacting protein) kinase complex, which is activated by other proteins such as the 9-1-1 complex, and subsequently phosphorylates and activates CHK1, leading to cell cycle arrest and consequent DNA repair, fork stabilization or replication start; 2) work as an R-loop (a tri-strand RNA-DNA hybrid) sensor, inducing the resolution of these structures by RNaseH1, and 3) unfold G-quadruplex structures with the help of the RecQ helicases BLM/WRN. Moreover, RPA can also promote DSB repair by Homologous Recombination (HR) and fork reversal by association with SMARCAL-1 (SWI/SNF-related, matrix-associated, actin-dependent regulator of chromatin, subfamily A-like 1). The other OB-fold containing protein working during replication stress is BRCA2, a tumor suppressor that plays an important role in DNA repair. It was recently described that BRCA2 could protect reversed replication forks from nuclease attack probably by recruiting and stabilizing RAD51 nucleofilament at the nascent strand. Components of the CST complexes also contain OB-fold domains, and due to their structural conservation, they are considered telomeric RPA-like proteins. They play important roles in telomere protection and maintenance in budding yeast. In humans, the CST complex does not present a telomere capping function but helps synthesize telomeric DNA at the lagging strand and mediates C-strand fill-in. However, some of the non-telomeric CST functions remain to be better understood. It was recently shown that it plays a role in active replication and at stalled replication forks. For example, CST was shown to facilitate re-initiation of DNA replication at repaired forks and dormant origins. CST can also be localized at the stalled replication fork and stabilize this structure by blocking the degradation of the nascent strand at the fork. Moreover, recent studies show intimate cooperation among the OB-fold proteins (RPA and BRCA2) and the CST complex to preserve DNA replication events and maintain genome stability.

## DNA Damage Repair and the Maintenance of Genome Stability

Cells have developed throughout evolution different mechanisms to repair most DNA lesions either endogenously generated or induced by exogenous agents. This played a key role in preserving their genomes across cell divisions since the accumulation of chromosomal mutations and aberrations has harmful consequences for the organisms, mainly by threatening the genome stability. The compilation of highly sophisticated and conserved mechanisms that ensure timely error correction or tolerance is inferred as DNA damage response (DDR).

Luna-Maldonado et al. summarized recent results in the literature demonstrating clear crosstalk between DDR and the Spindle Assembly Checkpoint (SAC) proteins to maintain genome stability and cell homeostasis. The review summarizes the roles of DDR proteins in mitosis and how SAC proteins regulate the response to DNA damage throughout the cell cycle. DDR provides DNA repair in a cascade manner, initiating damage recognition by sensors like PARP or DNA-PK. Subsequently, downstream signaling recruits damage transducers (e.g.: CHK1) and effectors whose activation depends on the phosphorylation of two major kinases, ATM (Ataxia Telangiectasia Mutated) and ATR (Ataxia Telangiectasia and Rad3 related), to initiate the repair. The SAC complex formed by the Mitotic Checkpoint Complex (MCC) proteins, CDC20, MPS1, and AURORA B, participate in mitosis by controlling the transition from metaphase to anaphase, ensuring correct chromosome segregation. According to Al-Jomah et al., Pds5A and Pds5B are good examples of this crosstalk between SAC and DDR. The depletion of one or both proteins can have different effects: phosphorylation of Chk1 with concomitant acetylation of Smc3 (a cohesin subunit), and inhibition of DNA replication and SAC activation. Of note, both are non-redundant but overlapping functions of Pds5A and Pds5B.

Among the different types of DNA lesions, DSBs are the most harmful to the cells since their processing and repair can cause insertion/deletions, loss of heterozygosity, chromosome translocations and rearrangements, resulting in cell death or tumorigenesis. However, as da Silva reported, DSBs formation is apparently crucial for the single-celled protozoa belonging to Trypanosomatidae family (e.g: *Trypanosoma brucei, Trypanosoma cruzi,* and *Leishmania* spp.), contributing to parasite evolution, survival, and adaptation to hosts and environmental barriers. In these organisms, DSBs are associated with antigenic variation (*T. brucei*), genetic exchange (*T. cruzi*), and genomic changes by gene copy number variation (*Leishmania* spp.). Curiously, in these parasites, most DSBs lesions are repaired by HR since the classical non-homologous end-joining (NHEJ) pathway is absent. Some species can also use microhomology-mediated end-joining (MMEJ) and single-strand annealing (SSA) to repair DSB lesions. However, in some circumstances that depend on the number and location of lesions, cell cycle phase, and cell repair capacity, DSB repair can also be disadvantageous, reducing parasite fitness leading to death or a dormancy state.

Different pathways rely on DNA protein kinases (DNA PKs) responsible for detecting the lesions and signaling to the correct pathway to initiate an appropriate DDR. Although conserved in model eukaryotes, DNA PKs present less conservation among trypanosomatids. They share partial functional redundancy with ATM, which was already identified in these parasites. Silva et al. compiled recent literature implicating ATR and ATM kinases in trypanosomatids DDR. They speculate about using known DNA PK inhibitors to identify their trypanosomes counterparts. However, a putative DNA PK homolog showing sequence conservation within its C-terminal kinase domain was identified in *Leishmania* spp. and *Crithidia* spp. ATM is less conserved than ATR in trypanosomatids, lacking important domains but preserving conservation at the C-terminus as ATR. In addition, the regulation of trypanosomatid ATM by phosphorylation is unclear and may even differ between related parasites. For example, depletion of ATM in *T. brucei* may be non-essential, and its inhibition in *Leishmania* spp. shows a moderate slowing of parasite proliferation with little perturbation of the cell cycle progression.

In contrast, ATR seems to be essential only in *T. brucei*. Authors speculate if trypanosomatids ATR, similar to other eukaryotes, would play a telomere function. It is already known that one of its partners, RPA, binds trypanosomatids telomeres and may regulate telomere homeostasis. ATR is also linked to damage accumulation within subtelomeres in *T. brucei*, regions of R-loop formation. Moreover, the investigation of Marin et al. showed that *T. brucei* ATR is also involved in DSB repair by HR, being necessary for the recruitment and upregulation of RAD51 for γH2A site. ATR is also involved in replication fork stalling and mediates intra-S and partial G1/S checkpoint responses. Thus, it plays a central role in signal transduction and is critical for orchestrating parasites DNA damage response. Rinaldi et al. summarized recent research about the new roles of ATM and ATR in protecting the genome by sensing aberrant R-loops. R-loops are a three-strand structure formed by an RNA-DNA hybrid where a single-stranded RNA molecule pairs with a single DNA strand displacing the second DNA strand. They contribute to the important cellular process and, in general, R-loops accumulate at highly transcribed regions containing repetitive sequences, such as the tRNA and rRNA loci. Several DNA repair pathways (e.g., HR and nucleotide excision repair–NER) contribute to R-loop regulation. Their abnormal formation can threaten genome stability, and it is known that there are clear interconnections between their regulatory mechanisms and the cellular response to either replication stress or DSBs. Costa-Silva et al. showed how topoisomerase 3α is engaged in HR repair and replication stress in *T. cruzi*. The authors analyzed the effects of TcTopo3α knockout (KO) in different developmental forms of this parasite. Slight growth alterations were observed in epimastigotes, whereas trypomastigotes showed reduced *in vitro* invasion capacity and amastigotes decreased cell proliferation. Curiously, in epimastigotes and amastigotes, the authors detected a high number of dormant cells. Interestingly, epimastigotes could not resume cell growth when knockout parasites were exposed to ionizing irradiation. Moreover, these parasites could not efficiently repair DNA damage when challenged with drugs that generate replication stress. One of these drugs, MMS, also induced telomere shortening in TcTopo3α KO parasites.

Using human fibroblasts, Russo et al. demonstrated a new role of an atypical protein tyrosine phosphatase, DUSP3, in the maintenance of genome stability. DUSP3 can associate with nucleophosmin (NPM) under genotoxic stress (e.g.: UV irradiation), leading to its dephosphorylation, affecting homooligomerization, its nucleolus-nucleoplasm translocation rate, and the subnuclear (re)localization of some protein partners. All these effects collectively culminate in increased stability, phosphorylation, and transcriptional activity of p53.

Using different immortalized human cells, Magalhães et al. demonstrated that RhoA, one of the Rho GTPases, plays a role in genome stability by regulating the repair of UV-induced DNA damage by NER. Rho GTPases belong to a small family of signaling molecules that are key mediators of diverse cellular and physiological processes such as cell division, migration, and invasion. They also act as pro-survival factors and are implicated in the regulation of components of DNA damage response since they show increased activity and expression when cells are exposed to different DNA damaging agents. The authors showed that cells displaying normal levels of active RhoA are more resistant to UV-promoted cell death than cells with RhoA loss of function, which accumulated in G1/S phases, and showed low survival rates and reduced cell proliferation. In addition, RhoA loss of function cells were hypersensitivity to UV effects in a NER-deficient background.

## Telomere Homeostasis: The Role of Proteins and TERRA Noncoding RNAs

Telomere regulation and the control of telomerase activity are of keen interest for the understanding of many biological features involved in tumorigenesis, aging, and the survival of eukaryotes.

Telomeres, the physical ends of linear eukaryotic chromosomes, are composed of repetitive DNA in double-stranded and single-stranded forms. The single-stranded protrusions at the 3′ end (3′ G-overhangs) are formed due to the inability of DNA polymerases to complete DNA replication at the end of the lagging strand. These terminal structures are associated with proteins (e.g.: in humans, represented by six-protein complexes known as shelterin, and the CST complex) and a long noncoding telomeric RNA (TERRA, Telomere Repeat containing RNA), whose transcription is originated at the C-strand subtelomeric region. Their orchestrated actions prevent telomeres from being recognized as DSBs, avoiding a local DDR. Also, these complexes protect chromosome termini from recombination, fusion, and degradation, ensuring genome stability and cell proliferation. Ackerson et al. reported how cells distinguish end protection from DSBs since they share many features and factors. They also compared and debated which pathway is employed to repair DSBs: HR or NHEJ. Finally, they reached a consensus that DSB repair choice and keeping telomeres protected are mutually exclusive events for the cell. Curiously, as previously mentioned, in *T. brucei*, DSBs are mainly repaired by HR since this organism lack cNHEJ repair pathway. Thus, *T. brucei* telomeres can be rearranged mainly by HR-dependent events, which commonly occur at the subtelomeric region and is one of the important pathways used by this parasite during antigenic variation. Other specific features about *T. brucei* telomeres are the fact that TERRA is transcribed by RNA polymerase I and only from the active VSG (Variant Surface Antigen)-adjacent telomere, where large truncations frequently occur. The high amount of TERRA transcription and TERRA R-loops formation at these active sites, in their turn, promote telomere instability by inducing DNA damage repair by HR, which increases VSG switching and hence the parasite ability to evade the immune system. A similar phenomenon happens when the parasite is depleted from some shelterin-like proteins, as reviewed by Bibo Li’s et al.

Liu et al. demonstrated that the depletion of CTC1 is very harmful to the cells. CTC1 is a component of the mammalian CST complex (CTC1, STN1, and TEN1) involved in restarting stalled telomeric replication fork and the C-strand fill-in synthesis. It was previously shown that the absence of CTC1 leads to defects in fork restart, and its mutation caused cancer-prone diseases (e.g.: coats plus or dyskeratosis congenita). Liu et al. figured out that the expression of CTC1 can be controlled by a miRNA (miR-376a). miR-376a overexpression induced telomere replication defect and resulted in telomere shortening and direct replicative telomere damage. Moreover, its high expression was associated with the deregulation of CTC1 and a poor outcome for patients with rectum adenocarcinoma.

It is a consensus that telomeres maintenance depends on many factors and circumstances. It was early known that due to the inability of DNA polymerases to complete replication at the ends of DNA lagging strand, noun as “the end replication problem,” chromosome ends can lose telomeric DNA in each cell division. The end replication problem is usually circumvented by a specialized reverse transcriptase named telomerase. Telomerase is a ribonucleoprotein (RNP) minimally composed of an RNA, TER (telomerase RNA), that contains the template sequence copied by the reverse transcriptase protein component (TERT) during telomere elongation. Telomerase activity and its access to telomeres are controlled in many ways, for example, by the telomeric heterochromatin and telomerase RNP protein subunits. However, telomere replication is completed by the canonical replication machinery, whose action can be hampered by the formation of secondary structures, such as t-loops. Therefore, numerous factors participate in efficient telomere maintenance by preventing local replication fork stalling or promoting the restart of a stalled replication fork at telomeres. Bonnell et al. provided an extensive discussion about the difficulties associated with the passage of the replication fork through telomeres in yeast and mammals. The authors showed that these organisms share conserved mechanisms to ensure complete telomere replication.

Telomerase can also engage *de novo* telomere synthesis/addition in a DSB site to avoid nucleolytic degradation and chromosome rearrangements. The *de novo* telomere addition can be artificially induced using telomeric tracts or spontaneously at TG-rich sequences near a DSB. These events were already observed in yeasts and ciliates during chromosome fragmentation, resulting in functional telomeres. In yeast, it is negatively regulated by a kinase (MEC1, the ortholog of ATM and ATR) or by the 5′-3′ helicase Pif1, as reviewed by Hoerr et al. These events are counteracted by telomerase acting at critically short telomeres.

The biogenesis of the telomerase RNP complex is another important issue involving telomeres maintenance and regulation. Savelyev et al. showed that PARP1 [Poly (ADP-ribose) polymerase 1], apart from regulating protein-protein and protein-nucleic acid interactions and many other processes, modulate the affinity of the H/ACA box GAR1 and DKC1 proteins for the TER component. The authors showed that PARylation influences proteins’ RNA-binding properties and alters telomerase activity and telomere length. Thus, PARP1 is probably involved with the assembly and stability of the telomerase RNP complex and may be a useful target for anticancer drug development.

Oliveira et al. demonstrated that *L. amazonensis* telomere length is naturally shorter in the infective forms, and telomerase activity is dependent on the temperature that parasites live in their specific host (insects or mammals). They showed that the inactivation of the chaperone HSP90 by a specific inhibitor (17AAG) disturbed parasite growth, induced cell cycle arrest at G2/M phases, inhibited telomerase activity, and caused telomere shortening in a time-dependent manner. Also, HSP90 co-IP with the TERT component agreeing in favor of HSP90 being a parasite telomerase component and a potential antiparasitic target. Chaperones are also important for the assembly and stability of the telomerase complex in yeast and humans, as previously shown.

Finally, Novo opened an interesting discussion about telomeres in pluripotent embryonic stem cells (mESCs). mESCs telomeres adopt a non-canonical, relaxed epigenetic state characterized by the low density of histone methylation and high TERRA expression. In contrast, telomeres shorten each cell division in somatic cells due to the absence of telomerase activity and low TERRA transcription. The specific mESCs nuclear environment likely adopts unique architecture and compartmentalization of the diverse molecules (RNA/proteins, chromatin, and other nuclear factors) relying on forming membraneless LLPS (liquid-liquid phase separation) condensates of different sizes and constitution. The LLPS is involved in many nuclear events and may mechanistically explain the simultaneous occurrence of distinct biochemical processes in the nucleus.

